# β-Sitosterol: Supercritical Carbon Dioxide Extraction from Sea Buckthorn (*Hippophae rhamnoides* L.) Seeds

**DOI:** 10.3390/ijms11041842

**Published:** 2010-04-22

**Authors:** Marie Sajfrtová, Ivana Ličková, Martina Wimmerová, Helena Sovová, Zdeněk Wimmer

**Affiliations:** 1 Institute of Chemical Process Fundamentals of the AS CR, v.v.i., Rozvojová 135/2, 165 00 Prague 6, Czech Republic; E-Mails: sajfrtova@icpf.cas.cz (M.S.); Lickova.Iva@seznam.cz (I.L.); sovova@icpf.cas.cz (H.S.); 2 Institute of Chemical Technology Prague, Technická 5, 166 28 Prague 6, Czech Republic; 3 Institute of Experimental Botany of the AS CR, v.v.i., Isotope Laboratory, Vídeňská 1083, 142 20 Prague 4, Czech Republic; E-Mail: wimmerova@biomed.cas.cz (M.W.)

**Keywords:** *Hippophae rhamnoides* L., sea buckthorn seed, fractionation in time, supercritical carbon dioxide, β-sitosterol

## Abstract

Supercritical fluid extraction represents an efficient and environmentally friendly technique for isolation of phytosterols from different plant sources. Sea buckthorn (*Hippophae rhamnoides* L.) seeds were extracted with supercritical carbon dioxide at pressures ranging from 15–60 MPa and temperatures of 40–80 °C. Oil and β-sitosterol yields were measured in the extraction course and compared with Soxhlet extraction with hexane. The average yield of β-sitosterol was 0.31 mg/g of seeds. The maximum concentration of β-sitosterol in the extract, 0.5% w/w, was achieved at 15 MPa, 40 °C, and a carbon dioxide consumption of 50 g/g of seeds. The extraction rate was maximal at 60 MPa and 40 °C. Both β-sitosterol yield and its concentration in the extract obtained with hexane were lower than with carbon dioxide.

## 1. Introduction

Plants are renewable sources of a large variety of natural products [1]. A number of them have already been proved to display pharmacologically important features, and are used or considered for application in treatment of serious diseases [2]. People have been using medicinal plants as medicaments, colorants, spices or as ingredients of agents applied in cosmetics for centuries. In recent years, the market has become overloaded by synthetically produced medicaments, which have become cheaper and easily available in high quantities. Nowadays, natural products showing medical activity are regarded as important and receive priority, because they mostly show higher biological effectiveness that is not accompanied by undesired effects.

Selection of a convenient isolation technology, as well as selection of an extraction solvent and the nature of the plant material used influence the product quality. Conventional methods of isolation of biologically active plant products from the natural sources are distillation, extraction, percolation or squeezing. To augment the product quality and the quantity of the active natural products, novel procedures and technologies have been investigated. Supercritical fluid extraction (SFE) technology has been receiving much attention. Its principle consists of extraction of a natural material with a fluid existing in its supercritical state under defined conditions (temperature and pressure). A separation of the extract from the solvent is mostly done by controlled pressure reduction. Considering carbon dioxide as a most frequent supercritical solvent, it should be stated that due to its non-polar nature it is convenient for extracting vegetable oils, essential oils, sterols, some vitamins and other natural products present in plant oils. The extraction rate can be relatively easily controlled by changing temperature and pressure in the extraction vessel; factors which influence the solvating power of supercritical carbon dioxide.

Generally, our attention has been focused on phytosterols and phytoecdysteroids, a family of steroid compounds, which has not yet received adequate attention, even though different phytosterols have already been successfully applied in cancer treatment. Phytosterols as natural components of vegetable oils have received particular attention because of their capability to lower serum cholesterol levels in humans [3,4] resulting in a significant reduction in the risk of heart disease. However, phytosterols have also been found to have anti-inflammatory, anti-bacterial, anti-ulcerative and anti-tumor properties [5] and therefore their contribution to the value of natural products as medicinally active nutraceuticals is obvious. The total sterol contents in the seeds of sea buckthorn (*Hippophae rhamnoides* L.) samples belonging to two major subspecies (*H. sinensis* and *H. rhamnoides*) from Finland and China were 1.2–1.8 mg/g and their contents in the extracted oils were 12–23 mg/g. β-Sitosterol constituted 57–76% of the seed sterols [6]. In another study, supercritical fluid extraction gave higher levels of sterols in oil from sea buckthorn seeds (16.4 mg/g of the oil for total sterols and 7.9 mg/g of the oil for β-sitosterol) than cold pressing and extraction with hexane [7].

Due to its low-polar nature, β-sitosterol is easily soluble in pure supercritical carbon dioxide. Using SFE technology, β-sitosterol has been extracted together with other medicinal components from a number of plant sources as sea buckthorn pomace [8], rice pollard [9], hibiscus seeds [10], common dandelion leaves [11], lovage leaves [12,13], saw palmetto leaves [14], chaste tree [15], loquat seeds [16] or from pot marigold or monk’s pepper tree [17]. SFE technology, applied to the extraction of β-sitosterol from hibiscus seeds [10] was carried at pressures of 15–45 MPa and temperatures of 40–60 °C. Even though increasing the pressure and the temperature have resulted in higher yields of the extracted plant oil and its required components, the temperature of 50 °C was recommended for the extraction due to the observation of higher rates of plant product degradation at temperatures exceeding 50 °C. The yield of β-sitosterol was optimal at 50 °C (2.39 mg/g of dry raw material). A positive effect of increasing pressure and temperature during SFE was also observed during common dandelion leaf extraction [11]. When applying a pressure range of 15–45 MPa and a temperature range of 35–65 °C, supercritical carbon dioxide extraction of 100 g of common dandelion leaves afforded between 3.2–4.0 g of extract, containing 3.1–3.7% of β-sitosterol. The maximum yield of β-sitosterol (1.23 mg/g of the raw material) was obtained at a pressure of 45 MPa and a temperature of 65 °C. The results of supercritical carbon dioxide extraction of hibiscus seeds and that of common dandelion leaves were compared with Soxhlet extraction of the plant material with hexane and ethanol. The yields of β-sitosterol obtained by the SFE and by the Soxhlet extraction with hexane were comparable. Application of ethanol in Soxhlet extraction resulted in a higher quantity of total extract, however, the content of β-sitosterol in the extract was lower by one order of magnitude, compared with its content in extracts obtained by the SFE technique.

We have already referred to articles [7,8], in which the authors dealt with the supercritical carbon dioxide extraction of phytosterols from different parts of the sea buckthorn (*H. rhamnoides* L.). In contrast to these studies, the objective of the present work was to examine the changes in the extraction yields of oil and β-sitosterol sea buckthorn seeds in the course of extraction. The specific objectives of the present investigation consisted of: (a) measuring extraction curves for total extract and β-sitosterol within the pressure range of 15–60 MPa, and the temperature range of 40–80 °C, (b) optimizing the SFE conditions with respect to the rate of extraction and its selectivity to β-sitosterol, and (c) comparing the SFE with the conventional Soxhlet extraction with hexane.

## 2. Results and Discussion

### 2.1. Course of the Extraction

#### 2.1.1. Total Extract

**Figure 1.**
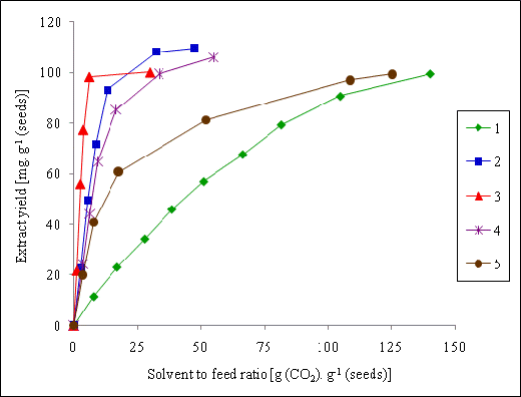
Extraction curves for the sea buckthorn seed oil. See Table 1 for the extraction conditions of experimental runs 1–5 (curve 1 to 5).

Figure 1 shows the extraction curves as the dependence of total extract yield on the quantity of the solvent passed. It is evident that the extraction conditions of the experimental runs 1 and 5 require a high consumption level of the solvent. This is due to the low solvent power of carbon dioxide related to its relatively low density (see Table 1). On the other hand, the conditions of the experimental run 3, where the pressure is set to 60 MPa, were optimal SFE conditions for fast extraction with low consumption of carbon dioxide.

#### 2.1.2. β-Sitosterol

**Figure 2.**
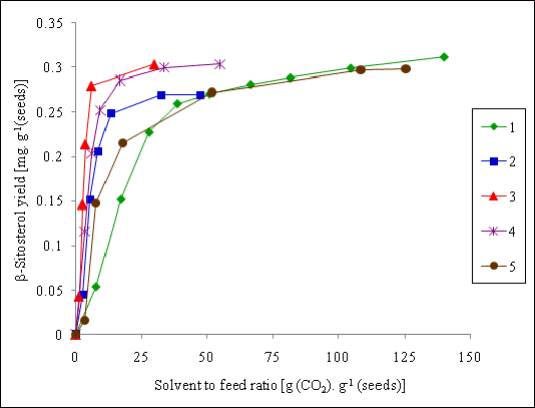
Extraction curves for β-sitosterol. For the extraction conditions of run 1–5 (curve 1 to 5) refer to Table 1.

Figure 2 shows the dependence of the β-sitosterol yield on the quantity of the solvent passed. With respect to the solvent consumption and to the extraction rate, the most convenient SFE conditions for the extraction of β-sitosterol were those of the experimental run 3 (pressure 60 MPa, temperature 40 °C), similarly to the optimal conditions for the total extract.

**Figure 3.**
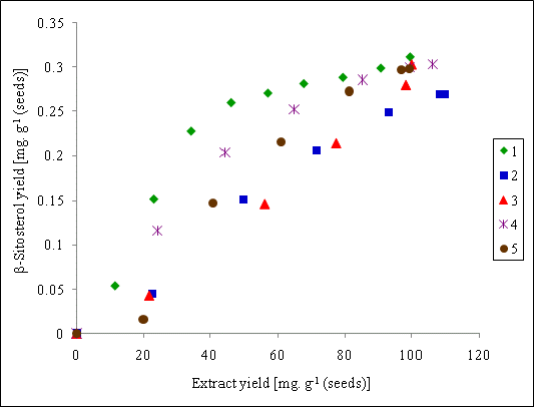
Relationship between β-sitosterol yield and total extract yields. For the extraction conditions of run 1–5 (curve 1 to 5) refer to Table 1.

However, when the objective of optimization of extraction conditions is to maximize the concentration of β-sitosterol in the extract, the result is different. This quantity is represented by the slopes of the curves in Figure 3, in which the yield of β-sitosterol is plotted against the total yield of extraction. It is evident that the most convenient extraction conditions were those of the run 1 (pressure 15 MPa, temperature 40 °C) and that the extraction should be stopped after passing over approximately 50 g of carbon dioxide per 1 g of extracted raw material when the extraction of β-sitosterol is almost complete. A continued SFE would result in a “dilution” by undesired products. Interestingly, the experimental curves in Figure 3 exhibit an initial shift corresponding to a delay of the start of β-sitosterol extraction. It can be explained by an initial extraction of highly soluble plant products that compete with β-sitosterol for the limited solvent capacity.

### 2.2. Comparison of the SFE with Conventional Extraction

The effectiveness of supercritical carbon dioxide extraction and Soxhlet extraction with hexane was compared. The yield of oil was slightly lower for SFE: 121.1 mg/g of the raw material using hexane and 99.3–109.3 mg/g of the raw material with supercritical carbon dioxide in dependence on the extraction conditions (cf. Figure 1).

The average yield of β-sitosterol reached 0.31 mg/g of raw material when supercritical carbon dioxide was used. On the other hand, conventional Soxhlet extraction with hexane afforded β-sitosterol in a quantity of 0.123 mg/g of raw material. When comparing the extractions used for component selectivity, the supercritical carbon dioxide extraction proved its advantage, providing up to five-times higher concentration of β-sitosterol in the extract (0.50% w/w) over the conventional Soxhlet extraction with hexane (0.10% w/w). The reason may undoubtedly be seen in the lower selectivity of hexane and in its high extraction temperature combined with the effect of atmospheric oxygen, which probably caused partial degradation of β-sitosterol. Thus, the supercritical carbon dioxide extraction has been proven to be a convenient extraction technique for obtaining a concentrated extract of β-sitosterol.

## 3. Experimental Section

### 3.1. Material

The berries of sea buckthorn were harvested from plants grown in the Czech Republic. Juice was expressed from the berries, the residue (a pulp and a seed mixture) was dried at room temperature, and the seeds were manually separated from the pulp and stored. The seeds were ground in a coffee mill prior to the extraction. The Sauter mean diameter of seed particles was 0.26 mm, as determined by sieve analysis.

Carbon dioxide of purity >99.9% was purchased from Linde Gas. Methanol and 2-propanol for HPLC (Chromservis, CR) were used as mobile phase for chemical analysis. The chromatographic standard of β-sitosterol (≥97%) was purchased from Sigma (Sigma-Aldrich, Germany). Hexane (>95%, Lach-Ner, CR) was used as a solvent for the Soxhlet extraction.

### 3.2. Supercritical Carbon Dioxide Extraction

**Figure 4.**
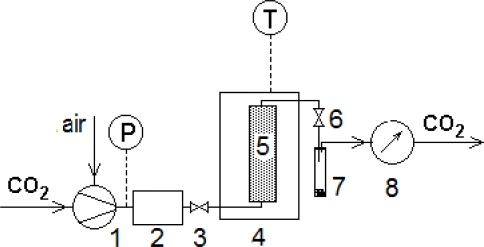
A scheme of the experimental device: 1-high pressure pump, 2-controlling unit, 3-input valve for carbon dioxide, 4-air-conditioned box, 5-extraction column, 6-expansion valve, 7-vial containing the extract, 8-flow meter.

The SFE experiments were carried out in an apparatus Spe-ed SFE (Applied Separations, USA; for its scheme see Figure 4). The extraction column (volume 25 mL; inner diameter 14 mm) was filled with 7 g of plant particles placed between layers of glass beads serving as solvent flow distributors. The extractor was connected to the equipment with stainless steel capillaries and placed into the air-conditioned box. Before the extraction procedure started, the extractor was filled with carbon dioxide under the given pressure, and the conditions in the extraction column were allowed to set for 15 min to achieve an equilibrium state between the solvent and a solute. Carbon dioxide was sucked from a pressure container using a high-pressure pump cooled by water to 5 °C. Supercritical carbon dioxide entered the lower end of the extraction column. The solution flowing from its upper end expanded to the ambient pressure in a heated micrometer valve and the extract fractions were collected in empty glass traps at the ambient temperature. The quantity of the gaseous solvent leaving the trap was measured using a gas meter. The samples were weighed and stored in freezer for chemical analysis.

The carbon dioxide flow rate was adjusted to 0.8 g/min. Because the total quantity of carbon dioxide passed through the extraction column was set to approximately 60 g/g of raw plant material, the extraction time was about 9 h. The extraction pressure varied from 15 to 60 MPa, and the extraction temperature was set within the range of 40–80 °C (see Table 1).

### 3.3. Soxhlet Extraction

Dry plant material (10 g) was extracted with hexane (250 mL) in a Soxhlet apparatus for 7 h. The solvent was removed from the extract using a rotary evaporator; the extract was weighed and stored for analysis.

### 3.4. HPLC Analysis

A reverse phase HPLC was used for quantitative analysis of β-sitosterol in the extracts. The HPLC apparatus consisted of pumps Chrom SDS 150s and 15s (Watrex, CR), an evaporative light scattering detector (ELSD 2100; Polymer Laboratories, USA), and a UV-DAD detector (Thermo, Finnigan, Spectra System UV 6000 LP, USA). Two analytical columns were used in tandem: column 1 [Nucleosil C18 5 μm, 250 x 4.6 mm i.d. (Macherey-Nagel)] and column 2 [Biospher C18 5 μm, 250 × 4.6 mm i.d. (Labio, CR)]. The extracts were analyzed using methanol / 2-propanol (4/1, v/v; isocratic elution mode) as mobile phase at a flow rate of 0.8 mL/min. β-Sitosterol was detected by ELSD, and the chromatogram was simultaneously monitored by UV at 210 nm. The external standard calibration method was used for quantification of β-sitosterol content.

## 4. Conclusions

The SFE conditions were optimized for the yield and the composition of extract from sea buckthorn seeds. The maximum concentration of β-sitosterol in the extract was achieved at a pressure of 15 MPa, a temperature of 40 °C, and with carbon dioxide consumption lower than 50 g/g of sea buckthorn seeds. The maximum extraction rate was at the maximum pressure used (60 MPa) at 40 °C. The SFE performance was compared with that of the Soxhlet extraction with hexane. The total extraction yields were comparable for both methods; however the yield of the target β-sitosterol was up to five-times higher for the SFE technique. The SFE technique was proved to be optimal for the extraction and isolation of β-sitosterol from the sea buckthorn seed oil, mainly because of the high concentration of the target product in the extract.

## Figures and Tables

**Table 1.** A summary of the extraction conditions and the calculated carbon dioxide density^a^ for the five experimental runs.

**Run**	**p [MP]**	**T [°C]**	**ρ_CO2_ [kg.m^3^]**
1	15	40	781.2
2	30	40	910.6
3	60	40	1020.5
4	30	60	830.5
5	30	80	746.1

a The density was calculated according to the Altunin-Gadetski equation [18].
